# A Congenital Quadricuspid Aortic Valve Associated With Aortic and Mitral Regurgitation: Case Report and Literature Review

**DOI:** 10.7759/cureus.12986

**Published:** 2021-01-29

**Authors:** Shahrad Shadman, Mohammad Sadegh Asadi, Soroush Nomigolzar, Mohammad Sarfarazi

**Affiliations:** 1 Internal Medicine, University of Maryland Capital Region Health, Cheverly, USA; 2 Cardiovascular Disease, Howard University Hospital, Washington, D.C., USA; 3 Clinical Foundations, Ross University School of Medicine, Bridgetown, BRB; 4 Cardiovascular Diseases, University of Maryland Capital Region Health, Cheverly, USA

**Keywords:** aortic valve insufficiency, congenital heart disease, aortic valve, cardiac surgical procedures, heart valve diseases, quadricuspid aortic valve, aortic regurgitation, case report, echocardiography - heart failure - valvular heart disease, congestive heart failure

## Abstract

Quadricuspid aortic valve (QAV) is a rare congenital cardiac anomaly that commonly becomes symptomatic in the fifth or sixth decade of life and can present as an isolated finding or in association with other congenital cardiovascular abnormalities. Previously, QAV was mainly a postmortem or intraoperative diagnosis and data was very limited on its natural history, associated complications, and long-term outcomes. In recent decades, however, there has been an increase in the reported cases of QAV, considering the advances in the diagnostic modalities such as transthoracic echocardiography (TTE), transesophageal echocardiography (TEE), and cardiac magnetic resonance (CMR). In this article, we present a case of a congenital QAV associated with aortic regurgitation as well as briefly review the QAV classification systems, treatment options, and long-term outcomes.

A 48-year-old woman with a significant past medical history (PMH) of hypertension and coronary artery disease presented with shortness of breath, chest pain, and orthopnea for two to three weeks. The patient’s TTE showed severe aortic regurgitation with significant compromise in cardiac output that could not be otherwise explained. Subsequently, her TEE revealed QAV as the underlying source of these findings.

Although the diagnosis of QAV could be very challenging, it is crucial to be considered when evaluating a patient with inscrutable progressive aortic regurgitation. Today, as a result of technological advancement, QAV is being diagnosed more accurately and promptly. Since there are no universal guidelines defined for this cardiac anomaly, regular follow-up with these patients is imperative to monitor for early signs of valvular compromise and to treat accordingly through medical and surgical interventions.

## Introduction

The quadricuspid aortic valve (QAV) is a rare congenital cardiac anomaly with an estimated frequency of <0.05% [1]. Previously, QAV was mainly a postmortem or intraoperative diagnosis and data was very limited on its natural history, associated complications, and long-term outcomes. In recent decades, however, there has been an increase in the reported cases of QAV, considering the advances in the diagnostic modalities such as transthoracic echocardiography (TTE), transesophageal echocardiography (TEE), and cardiac magnetic resonance (CMR). QAV commonly becomes symptomatic in the fifth or sixth decade of life and the most common hemodynamic abnormality associated with this anomaly is aortic regurgitation (AR) [2]. There are two schemes that could be used to classify QAV, both of which are based on the aortic valve’s anatomical structure: the “Hurwitz & Roberts”, and the “Nakamura et al.” simplified classification.

Hurwitz and Roberts described seven variants of QAV, with type A and type B being the most common types. Diagnosis of QAV could be very challenging, but it is crucial to be considered when evaluating a patient with inscrutable progressive AR. We present a case of a 48-year-old woman who was found to have severe AR with significant compromise in cardiac output that could not be otherwise explained. We also briefly review the QAV classification systems, treatment options, and long-term outcomes.

## Case presentation

A 48-year-old woman was admitted to our department with chief complaints of shortness of breath, chest pain, and orthopnea for two to three weeks. The patient stated that she had a viral illness three weeks ago for which she was seen at an urgent care. Consequently, she got very short of breath when lying flat and had to use three pillows at nighttime. She also reported getting extremely short of breath with normal activities of daily living, such as bathing or wearing clothes. The patient’s medical history was significant for coronary artery disease status post stenting of left anterior descending (LAD) and right coronary artery (RCA), in addition to hypertension that did not require medication.

**Figure 1:**
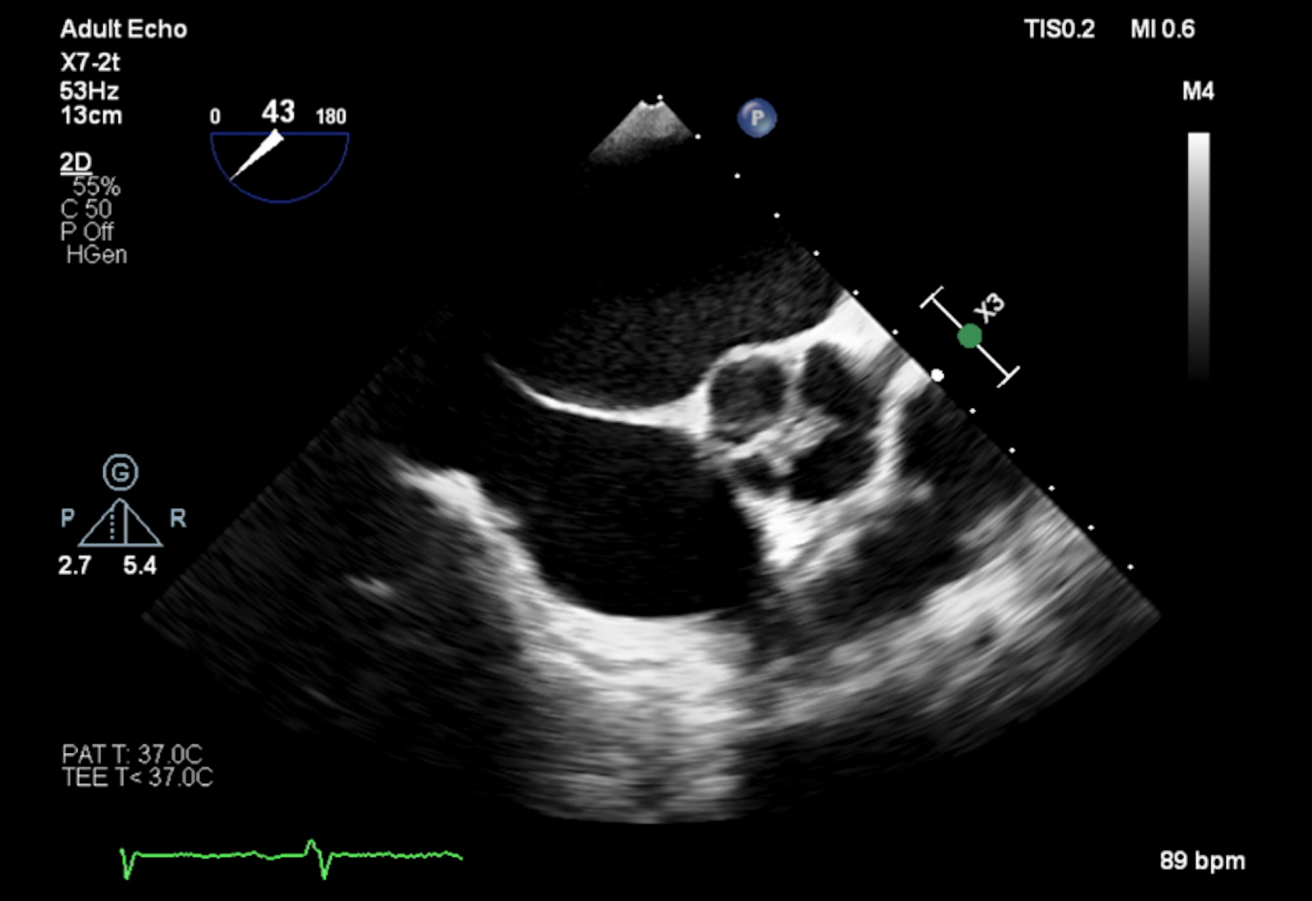
Transesophageal echocardiogram The TEE showing an aortic valve with four cusps. This QAV is classified as “Hurwitz and Roberts” type B, with three cusps almost identical in size and one smaller cusp. QAV: Quadricuspid aortic valve; TEE: Transesophageal echocardiography.

Vital signs were normal with a blood pressure of 113/74 mmHg, pulse rate of 60/min, temperature of 36.7 C, and respiratory rate of 15 breaths/min with normal oxygen saturation of 99% on room air. Physical examination revealed a grade 3/6 diastolic murmur in the aortic and mitral valve areas, with regular heart rhythm. Her lungs were clear to auscultation on both sides, and no pedal edema was present. The patient was noted to have a distended jugular venous pulse (JVP) with the nadir of the venous column on inspiration greater than 10 cm H2O. Electrocardiogram was significant for first-degree AV block, incomplete left bundle branch block, and T-wave inversion in leads aVL, V5, and V6. Troponins were repeated three times and were insignificant for ischemic disease. A TTE showed a severe reduction in left ventricular ejection fraction (15-20%). The patient was also found to have severe mitral and AR without any stenosis. There was no evidence of other significant valvular abnormalities. A subsequent TEE confirmed a quadricuspid aortic valve with three nearly identical cusps and one smaller cusp (Figure 1). CT angiogram of the chest showed a marginally dilated ascending aorta with a diameter of 4 cm.

Considering her risk of sudden cardiac death and presenting symptoms of congestive heart failure (CHF), the decision was made to proceed with an automated implantable cardioverter-defibrillator (AICD) placement. Cardiothoracic surgery was consulted to evaluate the patient for surgical aortic valve replacement. The patient was scheduled for elective repair of the aortic valve, once medically optimized.

## Discussion

A normal aortic valve is trileaflet with all three cusps being similar in shape and size. Anatomical variations of the aortic valve have been documented which are unicuspid, bicuspid, and quadricuspid variations. The bicuspid aortic valve is the most common aortic valve anomaly, followed by the unicuspid valve. QAV is thought to be the least common variation, with an incidence of 0.00028-0.00033% in autopsy series, 0.0059-0.0065% for patients undergoing TTE and 0.05-1% for those receiving aortic valve replacements for AR [2]. Balington first reported QAV in 1862 when he incidentally found this anomaly during an autopsy [3]. Since then, there have been approximately 200 reported cases of QAV.

**Figure 2:**
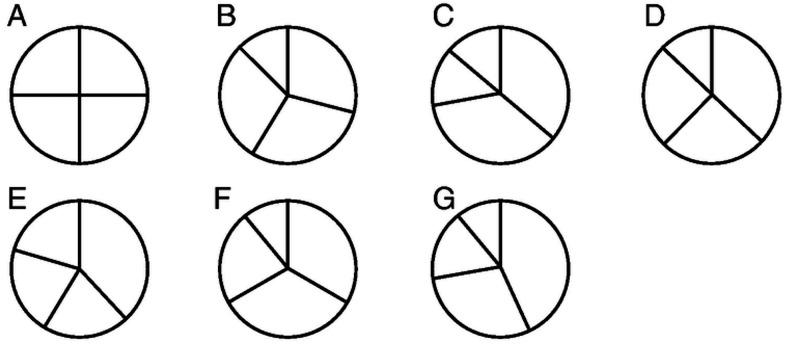
Hurwitz and Roberts Classification of the quadricuspid aortic valve Seven anatomical variations of quadricuspid aortic valve (QAV) were defined on the basis of size and symmetry of the aortic cusps. Adapted from Hurwitz and Roberts [4].

In 1973, Hurwitz and Roberts classified QAV into seven different groups based on leaflet size and the degree of cusp equality [4], as shown in Figure 2. According to this classification, type A is defined as four equal cusps, whereas type B is three equal cusps and one smaller cusp. Moreover, type C is two symmetrically large cusps and two equal smaller cusps, but type D is described as one large cusp, two intermediate cusps and one small cusp. Furthermore, type E is three cusps with equal size and one larger cusp, while type F is two equal-sized large cusps and two unequal smaller cusps. Finally, the seventh type of QAV in this classification is type G in which there are four unequal cusps. More recently, a new variation has been added to this classification. Type H comprises of two equal-sized small cusps and two unequal larger cusps [5]. The two most frequently reported variations are type A and Type B. Nakamura et al. simplified the classification system of QAV into type I to type IV, depending on the position of the supernumerary cusp relative to what is believed to be the anatomically normal cusps [6]. In this new classification system, types I and II are the same as the previously described types A and B, respectively. Our patient was found to have type B (or type II) QAV, with three equal-sized cusps and one smaller cusp.

QAV can present as an isolated anomaly or in association with several congenital cardiovascular abnormalities [7, 8]. In a literature review conducted in 2004, Tutarel found 186 cases of QAV, with only 154 cases detailing the functional status of QAV. Of these cases, 115 (74.7%) were found to have AR, while 13 cases (8.4%) had a combination of aortic stenosis (AS) and AR. Furthermore, only 0.7% of the cases reported AS alone [9]. A retrospective study that looked at 160,004 patients' cardiac imaging (TTE, TEE, CMR), identified eight cases of QAV (seven cases with AR and one with a combination of AR and AS) [10]. Other commonly associated abnormalities include ascending aortic dilation that was also seen in our patient, aortic root aneurysm, displacement of the coronary sinus and ostium, ventricular septal defect, patent ductus arteriosus, pulmonary stenosis, ruptured sinus of valsalva, and complete heart block [11-13]. Although it may not be as common as the aforementioned, a few cases of infective endocarditis have been reported in association with QAV [14-16]. It remains unclear whether QAV increases susceptibility to endocarditis due to the unequal distribution of shear stress. However, endocarditis prophylaxis is no longer recommended in patients with QAV.

In the past couple of years, there have been several reported cases of QAV with similar symptomatic presentations to our patient. A 48-year-old man presenting with progressive dyspnea on exertion, chest pressure, and palpitations, and a TEE showing QAV with severe AR underwent aortic valve replacement [17]. A 64-year-old woman with past medical history (PMH) of severe AR presenting with fatigue, dyspnea, and palpitations was found to have QAV and underwent valvular replacement [18]. A 34-year-old man presenting with progressive dyspnea was found to have QAV associated with moderate AR on TTE and underwent symptomatic medical treatment [19]. Unlike our patient who had QAV with severe aortic and mitral regurgitation and a dilated ascending aorta, these case reports portrayed patients with pure AR which is known to be the most prevalent anomaly associated with QAV. In all cases, cardiac imaging has proven to be necessary for making the diagnosis of QAV and associated cardiovascular anomalies and to determine the severity of abnormalities that lead to the patient's symptoms.

**Figure 3:**
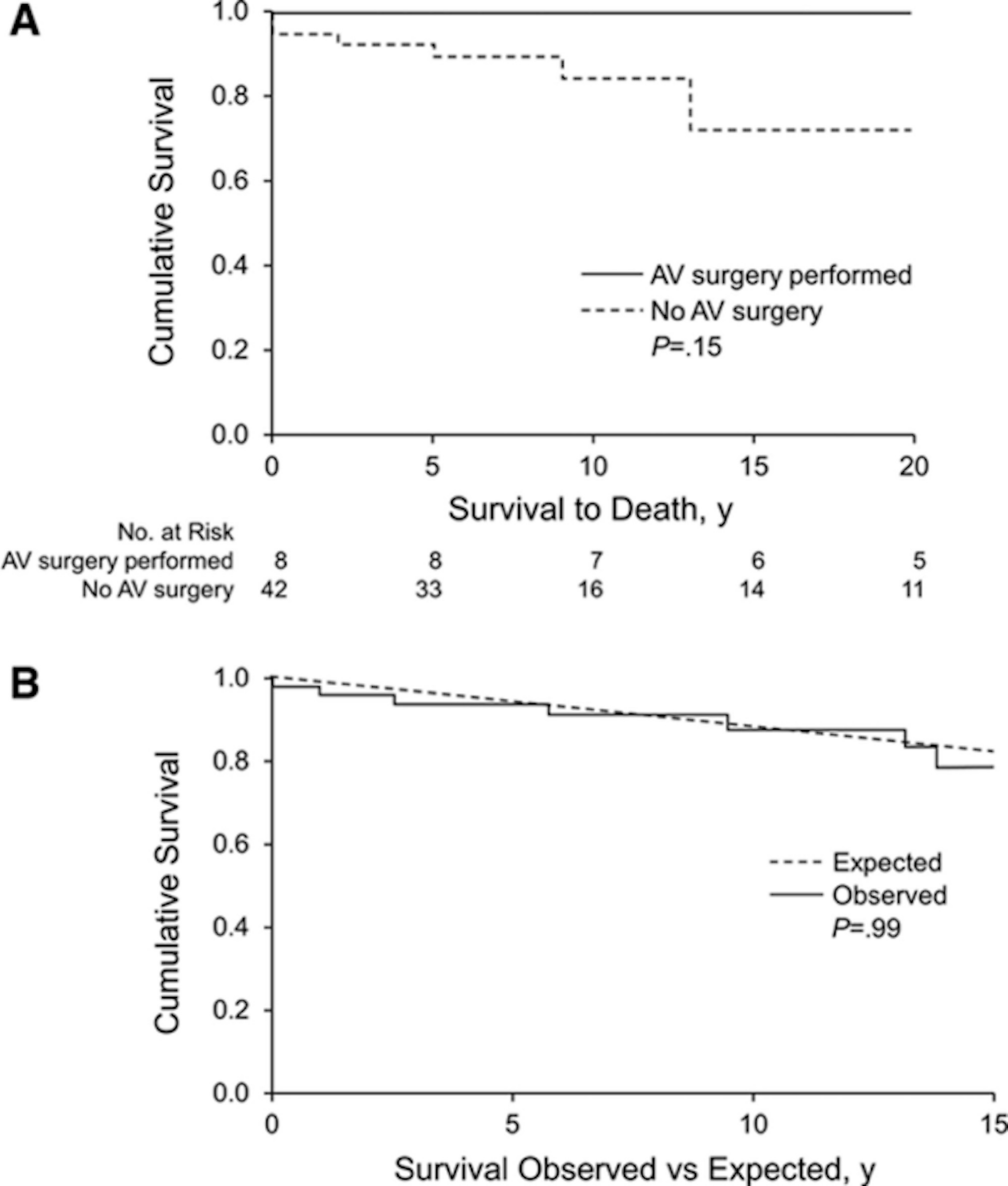
Kaplan-Meier survival curves The survival curve for patients with quadricuspid aortic valve (QAV) who underwent aortic valve surgery compared to those who did not (A) and survival curve for patients with QAV vs an otherwise healthy population (B). It can be concluded from the top graph (A) that aortic valve surgery did not prolong the life expectancy of patients with QAV compared to those who did not pursue surgery. The bottom graph (B) demonstrates that the observed survival of patients with QAV did not have a significant difference compared to the controlled group, defined as healthy individuals matched for age and sex. Adapted from Tsang et al. [1].

For symptomatic patients, treatment options include valve repair and replacement. Aortic valve surgery is the only definitive treatment in patients with QAV. AR remains the strongest indicator for surgery; however, the presence of severe AS or valvular dysfunction has also been reported as indications for surgery. Janssens et al. reported that 66.7% of QAV patients with AR required valve replacement [20]. However, valve replacement may not be a suitable option for most patients. Postoperative complications related to valve replacement include thromboembolism, prosthetic valve degeneration, complete heart block, endocarditis, and bleeding. Tsang et al. investigated the long-term outcomes of patients with QAV, in patients who proceeded with the aortic valve replacement compared to those who did not undergo this surgical treatment [1]. As depicted in Figure 3, there was no significant survival benefit for those patients that underwent valve surgery in comparison to those who proceeded nonsurgically.

## Conclusions

In summary, we presented a case of a quadricuspid aortic valve with severe aortic regurgitation. Our patient presented with symptoms relating to congestive heart failure (NYHA III-IV) likely due to severe aortic regurgitation (secondary to QAV) and severe mitral regurgitation. She was also found to have mild dilation of the ascending aorta. QAV is an extremely rare congenital cardiac anomaly, now being diagnosed more accurately and promptly as a result of advancement in diagnostic modalities. About 25% of patients with QAV require surgery, either repairing or replacing the aortic valve in patients with severe aortic valvular dysfunction. Recent studies have shown that tricuspidalization is a preferred repair option. Since there are no universal guidelines defined for QAV, regular follow-up with these patients is imperative to monitor for early signs of valvular compromise.
